# Contemporary Cultural Trade of Lion Body Parts

**DOI:** 10.3390/ani12223169

**Published:** 2022-11-16

**Authors:** Peter G. R. Coals, Nolwazi S. Mbongwa, Vincent N. Naude, Vivienne L. Williams

**Affiliations:** 1 School of Animal, Plant & Environmental Sciences, University of the Witwatersrand, Johannesburg 2000, South Africa; 2 Institute for Communities and Wildlife in Africa, University of Cape Town, Rondebosch 7701, South Africa; 3 Department of Conservation Ecology and Entomology, University of Stellenbosch, Matieland 7602, South Africa

**Keywords:** ancestral spirits, captive-breeding, *Panthera leo*, conservation policy, *muthi*, social justice, stakeholder engagement, sustainable use, traditional medicine, One Health, illegal wildlife trade

## Abstract

**Simple Summary:**

Cultural and traditional practices play a substantial but generally underestimated and poorly understood role in the use of wildlife. While the trade in traditional medicine or ‘*muthi*’ is a dynamic network of stakeholders, for this study we focused on commercial traders and traditional healers. We investigated various aspects of trade in lion parts through a semi-structured questionnaire, focusing on cultural applications, demand, trade dynamics, sources, and supply. In order to enhance our understanding of the challenges of product access and equitable use, we explored various motivations behind use (e.g., cultural and livelihood), perceptions of sustainability, and welcomed any participant-proposed initiatives to conserve lions. Our results indicated that lions were used symbolically for their power, strength, and nobility, and in rituals of healer training. Most participants believed lion populations to be declining and offered solutions to maintain lion product supplies. We highlight the importance of understanding cultural influence in the use of wildlife to ensure that all stakeholder voices are included in the development and implementation of conservation initiatives.

**Abstract:**

Trade in lion parts associated with cultural and traditional use is poorly understood. Here we sought to better understand the role and use of lion body parts in the commercial traditional medicine (*muthi*) trade of South Africa. In 2019 we conducted a semi-structured questionnaire survey of *muthi* traders (*n* = 10) and traditional healers (*n* = 20) which explored the significance and symbolism of lions, traded parts and preferences, sources and supply of lion parts, and perceived sustainability of lion derivatives in the South African *muthi* trade. Our results suggest a cultural importance of lion associated with the *umndawu* ancestral spirit in particular, as well as in the training and practice of cultural–spiritual healers. Lion paws and parts thereof were most frequently reported as sold by traders and demanded by healers, correlating with recent trends in body-part removals from lion mortalities. Respondents indicated that lion parts were obtained from a variety of sources including wild lion populations in neighbouring countries and captive-breeding farms. Our findings are discussed relative to current concerns in lion conservation and highlight a need for further understanding of the traditional medicine complex, the influence that ancestral spirits have on lion body-part trade, and increased engagement with traditional medicine stakeholders.

## 1. Introduction

Wild lions (*Panthera leo*) are experiencing widespread population declines in all but four African countries (i.e., Botswana, Namibia, South Africa, and Zimbabwe), having disappeared from ~92% of their historic range and leaving an estimated 23,000–39,000 adult individuals remaining in the wild [1,2]. Established threats to lions include prey depletion, livestock encroachment, illegal killing due to a real or perceived conflict over livestock, and habitat transformation [1,3]. In addition, there are contemporary concerns that consumptive use and trade in lion derivatives poses a threat to some wild lion populations [4,5]—however, information is limited as to the extent of targeted killing of lions for these purposes [6]. 

Although bone trade to Southeast Asia has dominated considerations of lion body-part trade for a decade [5,7,8], within Southern Africa, lion derivatives are also acquired and traded domestically for cultural purposes, including: zootherapeutic or traditional medicines, clothing, ornamentation, bushmeat, and curios, involving intra-continental supply chains [5,7,9]. Despite the prevalence of domestic trade and use of lion body parts [10,11], ‘traditional’ and ‘local’ uses are typically over-looked and neglected as potential deterministic factors driving wild lion exploitation, poaching and even population decline in regions of low density [4,7,12], presumably because of cultural-political sensitivities linked to the policing of traditional practices [13,14]. 

In southern Africa, as in the rest of Africa, lion parts are selectively used for an array of cultural purposes in association with corresponding, but varied, cultural beliefs [10,15]. These parts are used and incorporated into, for example, ritual preparations, cultural regalia, and objects in divination sets [16,17,18,19]. Ethnographic records suggest that lions have occupied a role in symbolically ‘loaded’ southern African cultural traditions [20], including the practices of traditional medicine. The selection of animals for certain healing and ritual practices is frequently aligned with the philosophy of the Doctrine of Signatures, which accords symbolic meaning to wildlife products based on species’ attributes that can be transferred to humans [13,21,22,23]. Lions are associated with attributes of power, strength, and fear, as well as being used to denote status and respect amongst kings, chiefs, and traditional healers [7,16,23]. Despite a rich ethnographic literature concerning wildlife use in traditional and cultural practices, the dynamics of contemporary culturally motivated trade and use of lion body parts [24], especially for traditional medicine, in South Africa remain poorly communicated in the peer-reviewed conservation-focused literature. 

South Africa is a stronghold for lion populations and is home to some ~3500 wild lions [1,25,26]. This managed lion metapopulation network comprises free-roaming populations and those maintained in smaller fenced reserves and protected areas across South Africa’s provinces [26]. In addition to these wild and ‘managed-wild’ lion populations, South Africa has substantial numbers of captive-bred lions [27]. This controversial practice of lion ‘farming’ and associated industries of captive-bred or ‘canned’ trophy hunting linked to intercontinental lion bone trade throughout Southeast Asia have brought issues of lion trade to the fore in both conservation and public discourse [28]. Considerable debate has focused on South African lion farming, particularly on how supplies of captive-bred lion products may influence trade dynamics and impact the few remaining wild lion populations [3,5,7,28]. 

In response to a relative paucity of evidence-based research into trade in wild lion body parts, the International Union for Conservation of Nature (IUCN) World Conservation Congress (WCC) Resolution 059 (Marseille, 2020)—‘Combatting the illegal trade in lion body parts and derivatives’ [29] calls for further consideration of drivers of poaching, illegal trade in lion parts and understanding of value-driven incentives for lion conservation. In this pilot-study we explored the trade of lion body parts in the context of commercial *muthi* (i.e., traditional, or cultural medicine) markets, and use by traditional healers, in South Africa. To our knowledge, this survey is the first to specifically seek to understand traditional, cultural trade in lion parts in a contemporary *muthi* system. Whilst recent attempts have been made to understand uses of lion, they have been as a restricted part of wider consideration of species used for *umuthi* as part of a small sample size survey solely focused on traditional healers (*n* = 5; [15]).

We surveyed market traders (*n =* 10) and healers (*n =* 20) with the aim of elucidating: (i) the significance and symbolism of lions in contemporary cultural, medicinal, and ritual practices; (ii) the current demand and reasons for lion use and trade; (iii) the types of lion parts being traded; (iv) sources and suppliers, and potential substitutes for lion parts; and (v) stakeholder views on the sustainability of lion part trade and the future of lion conservation.

## 2. Materials and Methods

### 2.1. Ethics Statement

This research was approved by the University of Cape Town, Faculty of Science Research Ethics Committee (#FSREC77-2019). Participant consent to be involved in the study was sought in-line with Chilisa [30] and followed a tiered approach: permission to interview traders was first obtained from the appropriate market chairpersons, while the appropriate *Indunas* (i.e., headmen) and the chairpersons of professional healer organizations were consulted to obtain permission to interview healers. Potential participants were informed of the purpose of the study before the consent form was verbally explained to each candidate who agreed to participate, thereby obtaining their informed consent prior to interviews (Supplementary Document S1). Data were collected without identifiers and all respondents remained anonymous. Where respondents withheld or withdrew consent, interviews were terminated along with any data collected to that point.

### 2.2. Study Area

**Figure 1:**
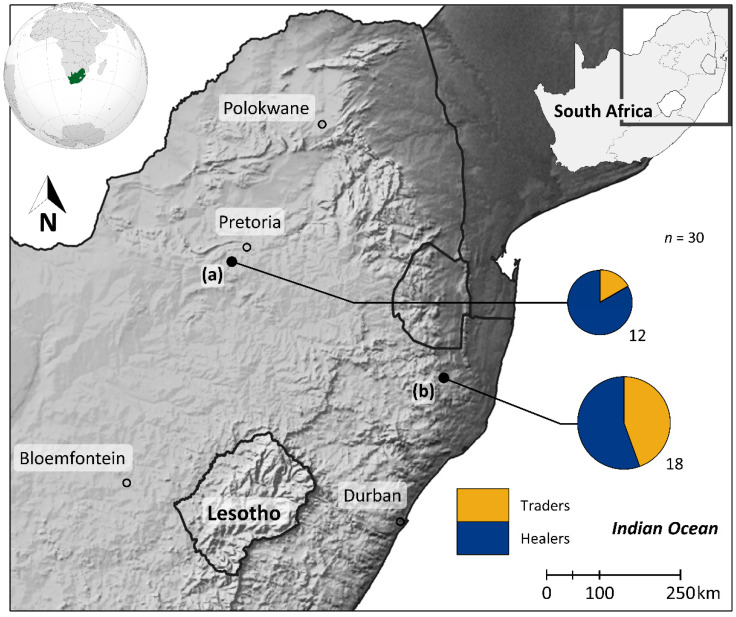
The study area in eastern South Africa where traditional *muthi* market traders (*n* = 10; yellow) and healers (*n* = 20; blue) were interviewed at (**a**) Faraday (*n*_traders_ = 2; *n*_healers_ = 10) and (**b**) Mona markets (*n*_traders_ = 8; *n*_healers_ = 10) between September and November 2019.

We focused on two of the preeminent (but not the only) traditional wildlife markets in South Africa where lion products were known to be traded ([21,31,32], pers. obs.). Respondents came from more than one trader market or healer association. Respondent selection followed a ‘snowball’ method whereby consenting participants shared the contact details of further potential participants. Interviews were conducted between September–November 2019 and were focused on Johannesburg (including the Faraday market) and Nongoma (including the Mona market) in the Gauteng (GP) and KwaZulu-Natal (KZN) provinces of South Africa, respectively (Figure 1). Faraday market, located in the central business district of Johannesburg, is considered the largest site for *muthi* (adjective) trade in South Africa at ± 400 traders [21,31]. In contrast, Mona market, in the Nongoma municipality of northern KZN is a regional market with ± 250 traders that only opens for a few days at the end of each month to coincide with pension payment days [32]. Though the Mona market does not function daily, it is widely used by rural healers and *umuthi* (noun) traders from surrounding urban areas. 

### 2.3. Study System

The traditional medicine or ‘*muthi*’ trade is a complex socio-economic system encompassing the supply and demand of wildlife products across a range of participating stakeholders [23,33,34,35]. This study focused on engaging with two major stakeholder designations to explore the cultural use and trade of lion parts in South Africa: informal commercial traders and traditional healers. Informal traders are typically involved in the commercial sale of products used in botanical and zootherapeutic cultural practices [21,23,36]. Hereafter, we define ‘traders’ as individuals with permanent or recurring traditional market stalls who commercially offered wildlife products for sale. The majority of traded wildlife in our study markets is known (i.e., from personal observation or experience) as being mainly, but not exclusively, traded in association with traditional medicine and other cultural uses such as traditional attire. These markets cater to both wholesale and end-consumer by supplying to *muthi* shops, traditional healers, and walk-in customers depending on the individual trader-based business model.

Traditional healers conduct sacred rituals as a fundamental part of their cultural-spiritual medicinal practice, wherein ancestors are considered divine messengers between the healer and a higher being [37,38,39,40]. Ancestors (e.g., *umndawu*, *amakhosi*, *abalozi*, *inyasa*, *idlozi*, *elimhophe*, *umndiki*, and *umnguni*) manifest in spirit during healing rituals and are specific to individual practitioners throughout South Africa [24,31,41,42]. Lions are known as *Ndau* in some ethnic groups across Southern Africa, thus the predominant ancestral spirit associated with lions is *umndawu*, a ‘go-between’ spirit which transcends ethnic groups [43]. Prophets (i.e., *moporofeti* [SeSotho], *umthandazi* [IsiZulu], *umprofethi* [IsiXhosa], and *muprofeta* [XiTsonga]), diviners (i.e., *selaodi* [SeSotho], *isangoma* [IsiZulu], *igqirha* [IsiXhosa], and *mungome* [XiTsonga]), and herbalists (i.e., *ngaka* [SeSotho], *inyanga* [IsiZulu], *ixhwele* [IsiXhosa], and *nyanga* [XiTsonga]) are the three most prominent and professionally recognised (Traditional Health Practitioners Act of South Africa; Act No. 35, Government Gazette, 2004) traditional healer groups in South Africa [44]. For the purposes of this study, we only interviewed diviners and herbalists (hereafter ‘healers’) as they are known to make use of wildlife products such as lion [5]. 

Diviners (*izangoma*) are usually chosen by ancestors (i.e., as part of a spiritual-belief system involving manifest ancestral will and guidance) and undergo training and initiation rituals (i.e., *ukuthwasa*—see [5] for details) that are uniquely tailored to the individual and guided by their ancestral spirits [44]. Diviners are then able to communicate directly with their ancestors to diagnose illness and prescribe the appropriate medication and treatment [24,45,46]. The ancestors of the patient may also be called upon to cater for individual needs and because individual family rites and rituals form part of the healing process [19,37,40,44]. 

Herbalists (*izinyanga*) undergo little spiritual training but develop their experience with wildlife products either with a traditional healer as a familial elder or are guided through visions and dreams on how to prescribe *umuthi* [37,47,48]. Herbalists are generally not entitled to consult with their ancestors in diagnoses [24,45], but may be shown how to prescribe the correct medicine once the patient’s ailment is known [49]. Owing to the ‘bridging’ nature of ancestral spirits between ethnic groups and traditions of practice we did not differentiate between tribal identities and ethnicities.

### 2.4. Survey Instrument

A semi-structured questionnaire, comprising 34 questions (*n*_traders_ = 11; *n*_healers_ = 10; *n*_both_ = 13), including a graphic with labelled lion parts, was used in this survey (Supplementary Document S2). Where interviews could not be face-to-face, they were conducted telephonically (*n*_healers_ = 5), and the questions and graphic were sent to participants via WhatsApp after acquiring informed consent. The questions posed (i.e., both closed and open-ended) focused on the conceptual value of lions and potential market dynamics behind the informal trade in lion products, as well as trader or user conscience-based views around sustainability and conservation practice. All answers were recorded verbatim.

### 2.5. Limitations

Sample size—Our sample size is limited to 10 traders and 20 healers. Owing to unforeseen external factors we were not able to sample further. We note that owing to our relatively small sample size any conclusions drawn must be taken to apply to the respondents in the system under consideration only. 

Veracity of ingredients and origins—It is possible that the origins and ingredients of lion products are more impenetrable and complex than our survey allowed for the exploration of. In particular, we did not explore how buyers determine whether products, which they acquire in the belief that they are from a lion, are genuine and how they vouched for their origin. Mechanisms to ensure that products are genuine, and that the provenance is known exist for buyers of wildlife products, for example through trusted supplier networks [50], however, the degree to which this is true in South Africa is untested. It is possible that such mechanisms are used in these markets, but we cannot be certain of the confidence that surveyed purchasers have in the veracity of lion products discussed. 

Timespans—Whilst we asked for respondents to indicate lion products that they bought and sold, we did not specify a timespan for such interactions. The questions were presented in the present tense (implying a degree of currency). However, we cannot be certain that respondents were not recalling past transactions in which they no longer participate.

Illegality and truthfulness—Respondents were not obliged or formally incentivised to answer questions. Questions were asked by a practicing member of the traditional healing community who is known and accepted in the study area (N.S.M.). We have no reason to suspect that answers provided were purposely misleading or untruthful. However, we do note that trade in lion products in South Africa requires Threatened Or Protected Species (TOPS) permits and formal registration in order to be conducted legally under the National Environmental Management: Biodiversity Act of 2004 (NEMBA No. 10, 2004). We did not question whether respondents had the requisite permits to possess and trade in lion products. Indeed, we consciously avoided discussions of legality with respondents, who remain anonymous. Given the potential for illegal practice, there is the possibility that respondents modified their responses. We used no sensitive questioning techniques in this study—future studies could usefully explore the use of such techniques in understanding the dynamics of *muthi* trade. 

Questioning—Questions provided in the survey tool were used as prompts to the questioner, but individual phrasing could be modified to better aid understanding and fit the flow of conversation. The interpretation of questions could thus be guided by the questioner. Whilst this might have insured that the topics answered were what was intended by the question it also introduces the possibility that responses were influenced by the questioner. In particular, we single out the questions H10, A3, and A8 as those which may be particularly vulnerable to issues of interpretation of the meaning surrounding ‘cultural value’ and ‘importance’. In-depth exploration of conceptualisations of cultural value by respondents was outside the scope of this study. However, it is likely that nuanced understanding of conceptions of value by stakeholders will be necessary in the development of a more complete picture of lion product usage in traditional medicine systems. N.S.M. is a traditional healer and carried out the questionnaire interviews; her integration with the traditional healing community made this study possible. However, there is the chance that respondent knowledge of N.S.M.’s understanding of traditional culture and practice influenced their responses, which we anticipate having positively impacted the information provided—but we cannot rule out respondents over-emphasising cultural importance. 

Our discussion of the results depends in part on unpublished and personal interpretation and explanation of cultural practices and terms—we endeavour to make clear, through indication of personal observation, when interpretation is provided outside of direct respondent quotation or the citable literature. 

### 2.6. Data Analyses

Data were visualised using a number of graphical interpretations. Significance and symbolism (of cultural value and use) used direct quotes from respondents to form word clouds, while other data were presented as bar and pie charts indicating the proportion of respondents, split into healers and traders, providing specific responses. Additionally, an incidence matrix of all lion body parts cited by each respondent for the respective questions answered was created, with regards to parts that were (i) sold by traders (*n* = 10), (ii) listed by traders as being in demand (*n* = 7), (iii) bought by healers for themselves (*n* = 20), (iv) bought by healers for their customers, and (v) bought by healers (i.e., combining iii and iv for each respondent; *n* = 20). Here, each row of the incidence matrix was summed to obtain the overall frequency of mention of the lion parts across the above-mentioned questions. Following Whiting et al. [51] and using the public-domain software EstimateS (Version 9.1.0; [52]), we calculated Sørenson’s similarity index (SSI) for incidence-based data [53]; the SSI quantified the degree of similarity of lion parts cited within and between respondent groups.

## 3. Results

### 3.1. Significance and Symbolism

**Figure 2:**
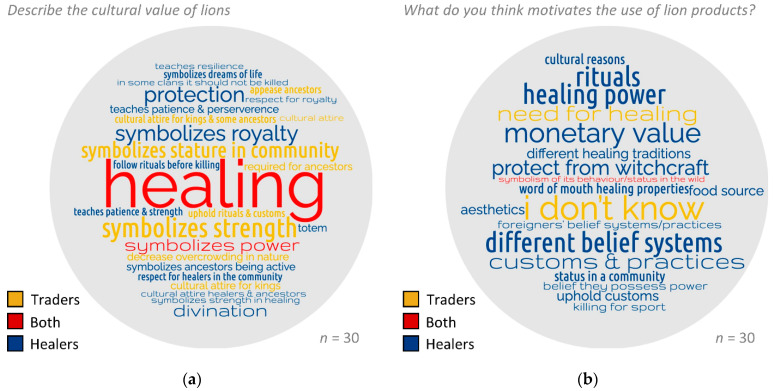
Significance and symbolism of lions and lion products from a survey (*n* = 30) of traditional *muthi* traders (*n* = 10) and healers (*n* = 20) in South Africa between August and September 2019. Responses are divided into those from traders only (yellow), both traders and healers (red), and healers only (blue). (**a**) A word cloud of descriptions for the cultural value of lions; (**b**) A word cloud of what is believed to motivate the use of lion products.

Amongst both traders and healers, healing was identified as the major cultural value of lion, in the context of *umuthi*, along with being considered a symbol of power (Figure 2a). Healers also highlighted associations with protection, strength, and patience, as well as symbolizing royalty. Traders spoke of community status, cultural attire, and ancestral linkages, and also mentioned the ecological role of lions (e.g., “reduce overcrowding in nature”)—an observation that was absent from healers (Figure 2a). 

There was little overlap in stated motivations for the use of lion products between healers and traders, but they agreed that the behaviour and status of lions in the wild is a symbolic motivator for using lion (i.e., lion is used for the attributes of wild lions; Figure 2b). Healers provided a diversity of answers relating to motivation for use with healing, monetary value, and use in cultural practices and rituals predominating responses. Amongst rarer responses were lion products as a source of food, and sport hunting (Figure 2b). Traders mostly said they did not know what motivates the use of lion products, except for the generic need for healing (Figure 2b). 

### 3.2. Requests, Preferences and ‘Demand’

Traders (*n* = 10) were questioned about customer requests for lion parts: five (50%) reported that customers requested parts from them, and five (50%) that they both recommended parts and received requests from customers. 

When questioned on whether lion parts were sold alone or mixed in with other medicines (i.e., other species), 50% (*n* = 5) of traders indicated that they sold lion parts alone (i.e., not mixed in with other species). One trader (10%) sold parts exclusively as mixed medicine (i.e., with both animals and plants), but they declined to provide details for which species. Forty percent (*n* = 4) of traders stated that they sold lion parts both as stand-alone items and mixed medicines; of those, half mixed lion parts with both animal and plant material (*n* = 2) and half declined to answer (*n* = 2). Only one trader (10%) provided details of which species they mixed lion with: leopard (*Panthera pardus*), cycads (*Encephalartos* spp.), and snakes (*Serpentes* spp.).

When traders were asked about the frequency of requests for lion parts (in the markets), a majority thought that requests were increasing (*n* = 6, 60%), 30% (*n* = 3) thought it was the same, and only 10% (*n* = 1) reported that they were uncertain. None said requests were decreasing, and healers (*n* = 20) were not asked this question. Reasons for increases in requests were suggested by two traders as: “changing healing traditions in South Africa” and “people wanting strength” (TR2), and the “influx of foreigners and increasing human population” (TR8). One trader did not know why requests for lion parts are increasing (TR1), and three did not answer. Of the three traders that said that the requests were the same, all provided the reason that they “still had the same clients requesting lion parts from them” (TR4, TR9, TR10).

**Figure 3:**
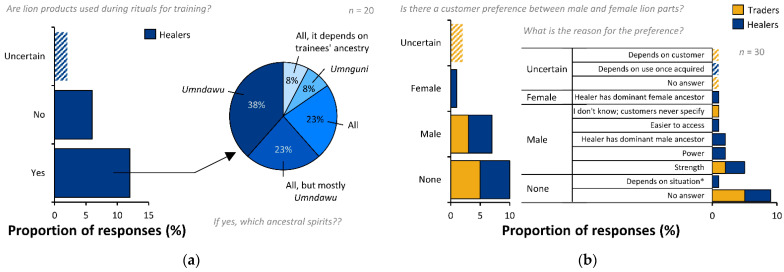
Use of lion parts during healer training and reported customer preference for male or female lion parts. (**a**) Use of lion products during healer training rituals and associated ancestral spirits. (**b**) Reported preference of customers of healers and traders for male or female lion parts, along with reasoning provided for that preference. * See full answer in text for respondent HE2.

When healers were asked whether lion products are used for rituals during training, a majority (*n* = 12, 60%) said “yes” (i.e., that parts were used for rituals during their training; Figure 3a). Of these, *umndawu* was the most frequently associated ancestral spirit, being mentioned specifically by eight (67%) healers—of which five (42%) healers stated *umndawu* as the only ancestral spirit, and three (25%) stated *umndawu* as the main spirit in conjunction with all ancestral spirits (Figure 3a). Seven (58%) healers said lion was important for all ancestral spirits. One healer declined to answer whether they used lion in training rituals, but stated they used lion for the *umnguni* ancestral spirit. The healers that were uncertain whether lions were used for training rituals provided no answer for associated ancestral spirits. Of the six (30%) healers who did not use lion in their training rituals the majority (*n* = 4, 67%) provided no answer, whilst one (HE15) said it “depended on the reason and ancestral calling”, and one (HE3) said “*esephothulu*”—for strength during and after graduation. 

Most healers bought lion products exclusively for themselves (*n* = 12, 60%), only one (5%) bought exclusively for customers, and the remainder bought for themselves and customers (*n* = 7, 35%). All healers (*n* = 20) answered the question—“If for yourself, what is it [lion] for?” and purposes given were: “in divination sets” (*n* = 8, 40%), “unspecified personal use” (*n* = 3, 15%), and for both “divination” as well as “other unspecified personal use” (*n* = 9, 45%). When traders and healers were questioned on whether customers prefer parts from male or female lions, half of the traders said their customers had no special preference (*n* = 5, 50%) and half the healers (*n* = 10, 50%) reported that their patients had no special preference for male or female lion (Figure 3b). Male lion was preferred by 30% (*n* = 3) of the traders and 35% (*n* = 7) of the healers. Female was preferred by one healer (*n* = 1; 5%), and the remainder of traders and healers were uncertain (healers: *n* = 2, 10%; traders: *n* = 2, 20%; Figure 3b). Healer reasons for preferring male lions were given as having a dominant male ancestor (*n* = 2) and the male having more power or strength (*n* = 5). Female lions were preferred by a healer with a dominant female ancestor (Figure 3b). Of those with no preference, the majority (*n* = 8), did not answer as to why. One said that there were different uses for male and female lions: “female for someone not to lose strength and power” [i.e., *engapheli amandla*], and male to ward-off evil spirits [i.e., “for initiates it should be male”] (HE2). Of the two healers that were uncertain, one declined to answer, and one said that “it depends on the use [of the product]”. Of the three traders that preferred male lion, two (TR2, TR4) stated that male was preferred because it has more strength, and one did not know “because customers did not specify” (TR8). All traders that indicated no preference (*n* = 5; 50%) declined to provide a reason and of the two traders that were uncertain about sex preference one (TR3) declined to respond and the other said that it depended on customer preference (TR10).

### 3.3. Trade

**Figure 4:**
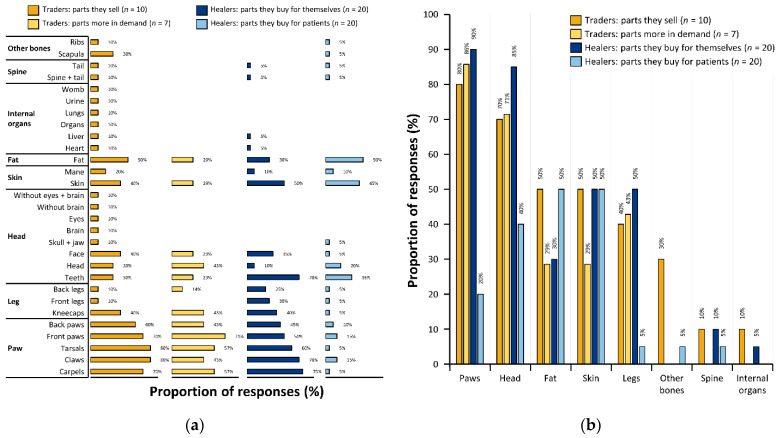
Trader and healer requests, preferences, and demand for lion derivatives. (**a**) Lion derivatives itemized on the questionnaire graphic and (i) the % traders that sell these derivatives (*n* = 10 traders); (ii) the % traders indicating that these derivatives are in more demand (*n* = 7); (iii) the % healers buying these derivatives for themselves to use in their practices (*n* = 20); and (iv) the % healers buying these derivatives for their patients/customers (*n* = 20). (**b**) Lion derivatives summarized as broad part types, and (i) the % traders that sell derivatives obtained from these part types (*n* = 10 traders); (ii) the % traders indicating that derivatives from these types are in more demand (*n* = 7); (iii) the % healers buying derivatives from these types for themselves to use in their practices (*n* = 20); and (iv) the % healers buying derivatives from these types for their patients/customers (*n* = 20). Paw part derivatives = claws, carpels, tarsals, and whole front and back paws. Head part derivatives = teeth, skull, jaw, face/muzzle, whole head, brains, and eyes. Fat = body fat. Skin derivatives = mane; full or partial skin and skin pieces. Leg part derivatives = kneecap, front and back legs (bones of whole). Other bones = ribs, scapula. Spine = spine, tail. Internal organ derivatives = heart, liver, lungs, womb, and urine.

Products derived from the paws predominated responses and were the items stated to be sold by the most traders: claws (*n* = 8, 80%); tarsals (*n* = 8, 80%); carpals (*n* = 7, 70%); front paws (*n* = 7, 70%); and back-paws (*n* = 6, 60%; Figure 4a). Fat was sold by 50% (*n* = 5) of traders, followed by kneecaps, faces and skin by 40% (*n* = 4) of traders (Figure 4a). The whole head, teeth and scapula featured with lesser proportions of traders (*n* = 3, 30%; Figure 4a). Other body parts, particularly soft parts, were rarer in responses, being sold only by a low proportion (*n* = 1, 10%) of traders (Figure 4a).

Traders stated that they sold an average of 8 ± 4 different body part types. Traders showed variable degrees of similarity between the parts they said they sold with 3.4 ± 2.9 parts the same (40% ± 32%, *n* = 10; Table 1). A few traders sold a wide range of parts, with one trader (TR5) listing all 19 body parts on the graphic—more than the other traders. However, the greater range of products were not products unique to that trader (i.e., though there were many different parts listed, they were not particularly uncommon). By contrast, one trader (TR6) sold an average number of body parts (eight different types), but these were self-listed ‘other’ parts (i.e., unnamed on the questionnaire graphic) that are uncommon in the markets and which the other traders did not sell (i.e., brains, eyes, heart, liver, lungs, womb, urine, and heads without soft tissues). 

Only seven traders chose to answer questions on the demand they experienced most for parts. Of this smaller sample, trader-reported demand largely matched those body parts sold by higher proportions of traders (Figure 4a). However, overall SSI was lower between traders as to which parts were in most demand compared to which parts were sold, listing only 1.8 ± 1.7 similar parts (35% ± 30%, *n* = 7) and the parts that they reported as being in demand showed only moderate similarity to the parts they sold (12, 59%; Table 1). Parts derived from paws were all recorded as being in demand (i.e., by the seven traders), along with kneecaps and back legs. Front paws were recorded as being the part most in demand (*n* = 5, 71% of traders) with tarsals and carpals second (both *n* = 4, 57%; Figure 4b). The teeth, head and face were also reported as being in demand amongst lesser numbers of respondents. 

Parts recorded as sold by traders were also recorded as being generally bought by healers too: overall, 20, 82%; for themselves 18, 77%; for patients 19, 79% (Table 1). Parts reported by traders as being in demand had a high degree of similarity to those healers reported buying (i.e., overall, 12, 75%; for themselves 12, 80%; for patients 12, 77%; Table 1). Parts from paws (*n* = 18, 90%) and head (*n* = 17, 85%) were frequently recorded as being bought by healers for themselves (Figure 3b). Teeth were sold by 30% (*n* = 3) of traders and purchased by 70% (*n* = 14) of healers. Fat was sold by 50% (n = 5) of traders and 30% (n = 6) of healers (for themselves) (Figure 3a). The majority of body parts sold by a low proportion of traders (*n* = 1, 10%) were not recorded as being purchased by any healers for themselves (i.e., womb, urine, lungs, internal organs, eyes, head without brain, head without brain and eyes, and brain; Figure 3a). Scapula was recorded in 30% (*n* = 3) of trader responses and did not feature in healer responses for themselves (Figure 3a). Scapula, skull and jaw, and ribs were reported as being bought by few healers (*n* = 1, 5%) for their patients (Figure 3a). Overall, healers reported that they purchased generally similar body parts for their patients that they bought for themselves, but no single item was bought by healers for their patients by a preponderance of respondents (Figure 3a,b). Teeth (*n* = 7, 35%), skin (*n* = 9, 45%), and fat (*n* = 10, 50%) predominated the body parts which healers reportedly bought for patients (Figure 3a). Only liver (*n* = 1, 5%) and heart (*n* = 1, 5%) were exclusively bought for healers themselves (Figure 3a). There was a high similarity between parts healers said they bought for patients and those they bought for themselves (87%). Healers showed slightly higher levels of Sørenson’s similarity for parts bought for patients (2.1 ± 1.1, 51% ± 30%, *n* = 11) than for themselves (3.0 ± 1.9, 46% ± 25%, *n* = 20; Table 1).

### 3.4. Source

**Figure 5:**
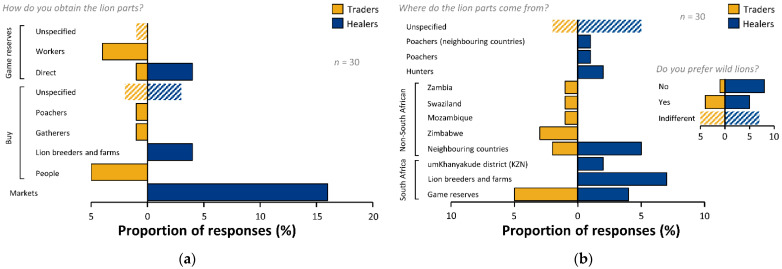
Acquisition and source of lion body parts: (**a**) how parts are obtained, and (**b**) where parts come from, including stated preference for wild-sourced lions.

Traders and healers showed marked differences in the sources from which they obtained lion parts (Figure 5a). Markets dominated healer responses (*n* = 16, 80%), with fewer responses relating to lion breeders and farms (*n* = 4, 20%) or direct from game reserves (*n* = 4, 20%; Figure 5a). Trader responses indicated that they bought parts from individuals: stating “people” as the highest proportion of responses (*n* = 5, 50%), with poachers (*n* = 1, 10%) and gatherers (*n* = 1, 10%) featuring in lower proportions of responses (Figure 5a). Traders also stated that they obtained lion parts directly from game farms, both from workers (*n* = 4, 40%) and directly (*n* = 1, 10%). 

Traders and healers provided detail as to sources of lion parts (Figure 5b). Game reserves in South Africa showed the highest proportion of responses amongst traders as a source of lion parts (*n* = 5, 50%). Traders made no mention of the type of people they obtained parts from (i.e., poachers or hunters), but provided detail on non-South African source countries with Zimbabwe showing the highest proportion of responses (*n* = 3, 30%) along with unspecified ‘neighbouring countries’ (*n* = 2, 20%; Figure 5b). Zambia, Eswatini (Swaziland) and Mozambique were mentioned by a trader (*n* = 1, 10%). Two traders (20%) did not know where their parts came from. Two healers chose not to participate in the source questions (*n* = 2, 10%). The remaining 18 healers identified a wider range of South African sources than traders, in addition to game farms (*n* = 4, 22.2%) they mentioned lion farms (*n* = 7, 38.8%), and the locale of Umkhanyakude District (KZN; *n* = 2, 11.1%). Healers also identified hunters (*n* = 2, 11.1%), poachers (*n* = 1, 5.6%), and poachers in neighbouring countries (*n* = 1, 5.6%) as persons who provided parts. Five (27.8%) healers stated that lion parts come from neighbouring countries, but none provided a specific location, and five (27.8%) did not know where parts came from.

When asked whether they preferred wild lions, 50% (*n* = 5) of traders indicated that they had no preference, one (TR4) stated: “As long as it is from an actual lion, it does not matter to me”, but the others provided no explanation. Only one trader (10%) stated that they did not prefer wild lions but provided no explanation. Four traders (40%) stated that they did prefer wild lions. Three traders (30%) provided reasons relating to the perceived strength of wild lions, and one trader (10%) stated that it was due to customer demand for wild lions. The greatest proportion of healers did not prefer wild lions (*n* = 8, 40%) but the majority of these healers (*n* = 5) did not explain their reasoning. One healer (HE15) cited the reason for not preferring wild as “background knowledge so that you can understand age, diet, medical history”. Two other healers (HE12 and HE13) stated that the product was the same regardless. Specific indifference was stated by 35% (*n* = 7) of healers. Two provided reasoning: “as long as it’s a lion it will perform the required duties” (HE19) and “works the same” (HE2). Fewer healers (*n* = 5, 25%) stated that they did prefer wild lions. All five provided reasoning: that wild was stronger, and that breeding, and husbandry changes the biology of the lion, e.g., “wild animals are still more pristine and exposed to the wild and their biology has not been altered” (HE3). 

### 3.5. Supply

**Figure 6:**
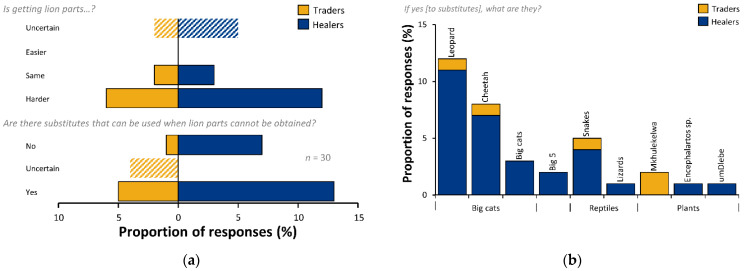
Trends in supply and substitutes for lion parts: (**a**) trends in supply, and existence of lion substitutes, and (**b**) respondent-suggested candidate substitutes for lion parts.

The highest proportions of responses by traders and healers indicated that they believed that it was becoming harder to get lion parts, lesser proportions stated that it was the same and none indicated that it was easier (Figure 6a). One trader, TR9, said “Sometimes I do not get what I need from suppliers”, and two traders (TR5 and TR6) said that lion was “very scarce”, while three did not comment. Three healers (HE14, HE17, HE18) stated that they could not get material from traders. One trader (HE10) stated that it was because lion was scarcer in the wild and three just said that it was scarcer (HE3, HE11, HE12).

In the event that lion parts were not available, traders and healers were asked if there were substitutes (Figure 6a). Trader opinions were dominated by “yes” (*n* = 5, 50%) and “uncertain” (*n* = 4, 40%), and only one trader (10%) said “no”. Healers had no uncertainty but were split in their opinions: thirty-five percent (*n* = 7, 35%) said there was no substitute but a greater proportion (*n* = 13, 65%) said that there was. Of those traders, five that said there were substitutes, and leopard (*n* = 1, 20%) and cheetah (*Acinonyx jubatus*; *n* = 1, 20%) were the big cats stated, along with snakes (*n* = 1, 20%), and *umkhulekelwa* (i.e., an unidentified plant; *n* = 2, 40%; Figure 6b). *Mkhulekelwa* was only mentioned by traders not healers. Healers stated a greater range of species: leopard (*n* = 11, 85%) and cheetah (*n* = 7, 54%) predominated, with snakes (*n* = 4, 31%), big cats (*n* = 3, 23%) and “the big five” (i.e., lion, leopard, African elephant [*Loxodonta africana*], black rhino [*Diceros bicornis*], and cape buffalo [*Syncerus caffer*]; *n* = 2, 15%) in lesser proportions of responses (Figure 6b). Lizards (*Lacertilia* spp.), *isigqiki-somkhovu* (*Encephalartos* spp.) and *umdlebe* (*Euphorbia cupularis* or *Pterocarpus* spp.) featured in the lowest proportions of healer responses (*n* = 1, 8% each). 

### 3.6. Sustainability

**Figure 7:**
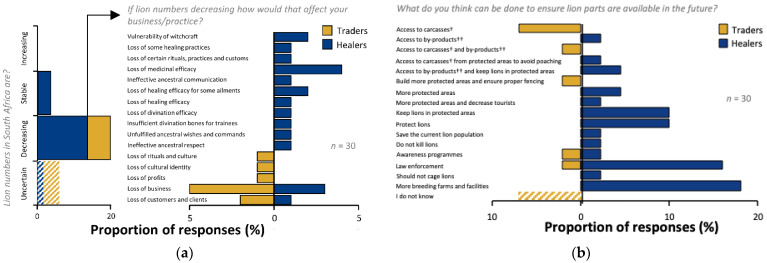
Perceptions of lion population status and sustainability in the trade in lion derivatives. (**a**) Perception of lion population trends and perceived effect of declining lion populations on business or practice, and (**b**) respondent-suggested solutions for ensuring lion parts are accessible in future. † Carcass refers to the material derived from a lion once destroyed, while †† by-products refer to non-destructively sourced material (e.g., urine, faeces, and mucus).

No traders or healers believed that lion numbers in South Africa were increasing (Figure 7a). The greatest proportion of traders (*n* = 6, 60%) indicated that they believed lion numbers to be decreasing, with the remainder (*n* = 4, 40%) uncertain. Likewise, 70% (*n* = 14) of healers believed lion numbers to be decreasing. Some healers (*n* = 4, 20%) believed lion numbers were stable, and 10% (*n* = 2) were uncertain. Traders were primarily concerned about the implications of decreasing lion numbers on their businesses and profits, and a small minority had concerns over loss of culture (Figure 7a). Healers were also concerned about loss of business, but also showed concerns regarding the efficacy of their practice and potential impacts on ancestral appeasement (Figure 7a). 

Traders and healers provided suggestions of what could be done to ensure access to lion parts in the future (Figure 7b). Trader responses primarily concerned access to carcasses and lion products—40% (*n* = 4) of traders stated, “give us carcasses”. Fewer mentioned lion conservation measures such as protected areas, law enforcement and, awareness. TR1 followed-up the desire for more protected areas with the proviso that they should have access for traders to buy “discarded” material. A desire for more breeding farms and similar facilities was expressed by 40% (*n* = 8) of healers. Protection of lion in a variety of ways was also expressed, with law enforcement being the most frequently cited measure (*n* = 7, 35%). No law enforcement responses by traders or healers corresponded to those who stated they sourced parts from poachers. However, a trader (TR10) who stated that they sourced parts from “gatherers in Zimbabwe” cited law enforcement as a means to ensure lion part availability in the future along with one healer who said that their lion parts came from “neighbouring countries” (HE7). 

## 4. Discussion

Here we provide an outline of the role of lion in contemporary *muthi* trade which may form the basis for further in-depth study. In particular, we note the diversity of cultural–spiritual traditions in South Africa, as well as those across the wider geographical southern African region. For that reason, we stress that our interpretations are limited to the specific system and stakeholders that we considered. Future work could usefully apply similar questions to the understanding of a wider range of African cultural–spiritual traditions.

### 4.1. Research Ethic

Our methodology represents a contextualised approach whereby we attempted to consider the sensitive cultural milieu of the respondents in the most appropriate and respectful way. To this end, our research also embodies *Ubuntu*. This sub-Saharan African philosophy, based on humanistic notions of connectivity, compassion, and virtue, may be applied in a ‘collectivist’ way of supporting and recognising knowledge collected in the context of the research participants’ values, world views and perceived realities [54]. Our research approach aimed to invite cognisance of ethical methods necessary to conduct investigations with stakeholders in a complex socio-cultural traditional medicine system. Use and trade of wildlife in this context is a particularly contentious subject, but the traditions of practice associated with lions are steeped in a rich ancestral and generational heritage that one must be mindful of at the outset and throughout such research. We choose *Ubuntu* as our holistic research ethic over other recent conceptualisations of inclusivity in conservation (e.g., [55]) for its rooting in an explicitly sub-Saharan African philosophy. By grounding inclusivity in an African philosophy, we intend that our research ethic be more fundamentally compatible with our participants as we are wary of the unintentional colonizing effect of methods of inclusion rooted in ‘western’ thought—though we note that *Ubuntu* is not dissimilar to many ‘western’ conceptions [54]. We caution against framings that represent traditionally motivated trade as inherently damaging to biodiversity [15]. Nevertheless, we acknowledge that our primary purpose in conducting this work was to gain information to inform lion conservation, whilst demonstrating a requirement for respect of traditional culture. Of particular importance is obtaining culturally appropriate permissions alongside research institution ethical oversight to ensure adequate safeguarding of the interests of human subjects of research [56]. We believe that instances where ethical approval and oversight has not been obtained (e.g., [15]) have the potential to jeopardise future interactions with stakeholders in *muthi* systems. We realise that a number of potentially conflicting stakeholder priorities are present in this complex system, including livelihoods, cultural practice, law enforcement, and wildlife conservation (see [27] for a linked lion trade system). We do not seek by invoking inclusivity to unduly favour one interest over another (e.g., cultural tradition over wildlife conservation). Rather, we highlight the need for detailed ethical exploration of this complex socio-cultural wildlife trade system to be made with consideration of socially just conservation principles [57].

### 4.2. Significance and Symbolism

Our results for the stated cultural value of lion are in line with long-established conceptions which relate to symbolic representations of strength, power, protection, and connotations of royalty [13,15,24,39]. We found general consensus between traders and healers regarding symbolism, indeed similar symbolism spans cultures beyond the African continent and geographic range of lions (e.g., [23,58]). In general, healers described the cultural value of lions in terms of symbolism and traders in terms of what could be done with them. These interpretations are unsurprising given the traders’ utilitarian, that is, commercial trade-based focus, as opposed to the more explicitly spiritual role of healers, who make extensive use of wildlife symbolism in their practice. Interpreting responses to the motivation for use of lion products is more challenging. We note that the primary knowledge-holders (i.e., healers) provided a diverse range of information which was focused on cultural ritual usage. It is also unsurprising to learn that monetary value was a frequent motivator for healers and the importance of traditional medicinal practice for stakeholder livelihoods has been noted previously in this trade system [15]. However, although the absolute contribution of lion sales to stakeholder incomes has not been assessed and is associated with significant complexities, we note that other species of plants and animals are likely to predominate [19,36,49]. 

### 4.3. Requests, Preferences and ‘Demand’

Although not trained as traditional healers, traders did appear to be in possession of a degree of cultural knowledge regarding use of lion. We did not test trader knowledge in further depth. However, future studies could work to better understand the feedback of knowledge and purchasing between traders and healers. It may also be that trader understanding of recommendations is also related to informing healers as to where they could source parts that the trader did not have in stock—we have repeatedly observed such (semi)cooperative behaviour amongst stakeholders in *muthi* markets (i.e., as part of our long-term involvement researching this system) in furtherance of healer traditional practice (pers. obs.). 

Traders sold lion parts both as stand-alone parts and medicinal mixes with other species. There are a wide range of uses of lion parts, therefore it is not necessarily the case that traders will sell lion parts exclusively for use as part of medicine as uses may encompass regalia and non-consumptive spiritual purposes [7]. For example, divination sets are used by most healers in their practice. A healer will typically compile their own divination set, based on items chosen by ancestral spirits, which will often include lion parts ([5,13,38,39]; pers. obs.). The common presence of lion in divination sets was supported by the responses we received to this questionnaire. Despite the widespread use of lion parts as components in healer tools of practice, such as divination sets, it is difficult to comment on the absolute demand that this may place on sources of lion as we did not consider the turnover and frequency of purchase of parts. We do note that whilst a healer may continue to add to their divination set throughout their life, such components are non-consumptive and are likely to rarely require replacement and are passed on from one generation to the other (pers. obs.). 

**Figure 8:**
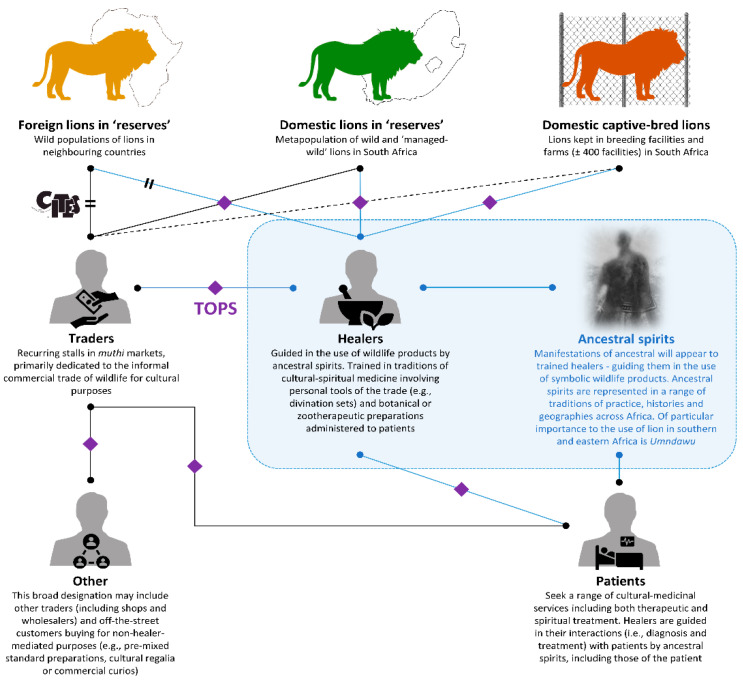
Traditional medicine (*muthi*) system diagram illustrating lion body-part trade and use linkages between stakeholders, with the inclusion of ancestral spirits (primarily, but not limited to, *umndawu*) as adjunct stakeholders and ‘gatekeepers’ mediating lion part use and trade. Solid lines indicate relationships revealed by responses to this survey; dotted lines represent relationships not revealed in responses to this survey but which have been previously recorded in the literature; blue lines indicate interactions gatekept and mediated by ancestral spirits; parallel bars indicate trade requiring CITES permits—in this system all trade is contrary to CITES; purple diamonds indicate trade and possession requiring a TOPS permit—not all trade in this system is necessarily in contravention of TOPS permitting.

Increasing requests for lion were suggested by some traders to be due to changing medicinal practices and foreign influence. The dynamic nature of South African traditional medicine has been previously observed; with cultural influences and people from across the African continent dictating product demand [13,38,39,59]. We could not probe the changing nature of lion use in depth, however a more detailed understanding of changing cultural uses may be necessary if attempting to predict demand and devise sustainable use interventions [13]. We also emphasize the role of ancestral spirits as drivers of lion part use and trade. Indeed, ancestral spirits may be conceived of as active stakeholders in the *muthi* trade system and appear to function as ‘gatekeepers’ that mediate the use of lion by healers (Figure 8). We advocate an understanding of traditional cultural practices whereby demand and use is influenced dynamically by ancestral spirits—more nuanced understanding of the role of ancestral spirits will likely lead to clearer pictures of the drivers of cultural–medicinal demand for lion.

### 4.4. Trade

We cannot speak to volumes or frequency of product in trade (see [21] for indications of trade volumes in *muthi* markets). However, our results do provide indications of the likely popularity and possible availability of certain lion parts in trade—though we note that interpretation is limited without indications of volume, frequency, and stock turn-over [60]. 

Parts derived from paws were the most frequently mentioned part types and therefore appear to have widespread popularity amongst traders (i.e., who trade lion) and healers. However, this may be as a result of more ready availability—we note that there are generally more paw-based products on an individual lion than other parts, with exceptions for other multiple or highly divisible parts such as fat, teeth, or skin—which were also featured in responses. Paw parts thus appear as close to ‘staple’ products as were identified in our sample. It did not appear that parts mentioned by fewer traders (i.e., ‘rarer’ parts in the survey) were necessarily traded by those traders who sold more different types of parts. The distribution of different types of lion parts between traders thus appeared uneven and patchy with lion part trade scattered amongst traders, and reasonably low levels of similarity between the parts traders said that they sold. It appears that whilst most traders stated that they sold generally ‘popular’ products (i.e., mainly paw-derived) other, less frequently mentioned parts are partitioned between different traders. Exploring the reasons behind such partitioning is beyond the scope of this study. Nevertheless, we highlight that although lion parts were partitioned amongst traders, this partitioning is broken down by (semi)cooperative referrals (pers. obs.). Indeed, there was a generally high degree of similarity between the parts traders stated that they sold, and the parts healers bought; thus, enforcing the role of traders as a primary source of lion parts for healers. 

Teeth showed a clear difference between the proportions of traders selling and healers buying: suggesting that a relatively low proportion of traders dominate the market in teeth, or that healers source teeth elsewhere—possibly lion farms and reserves. For many parts reported as sold by low proportions of traders, it is likely that the buyers of these products were not captured by the limited sample size of the survey. It is likely that a trader serves a greater number of healers (pers. obs.), and some parts would not be captured in a restricted sample of healers. It is also possible that healers are not the buyers for those body parts. Indeed, future studies could usefully seek to characterize and survey a fuller spectrum of buyers in *muthi* markets. 

No single part was reported as dominating healer purchases for their patients. The low proportions of responses for individual parts and the wide variety of parts purchased may be explained by healers tailoring their treatment to the individual requirement of the patient (pers. obs.), and supports a wide range of traditional medicinal uses for lion. Purchases for healers themselves is likely to be in connection with use in generic practice as opposed to targeted at individual patients, for example: divination sets used repeatedly by the healer are likely to contain lion paw bones (pers. obs.). We therefore suggest that some lion parts may be considered as more central to traditional cultural–medicinal practice, and that these are predominantly paw and head parts which healers use as components of their personal tools of the craft. Indeed, demand for parts was recorded to be primarily for paw, leg, and head products, and largely corresponded with the parts sold by higher proportions of traders. Body parts recorded as being in demand show similarities to parts most frequently removed from wild lions [61], notably: paws, claws, and head [4], and our results indicate that lion parts in South African *muthi* markets are likely to be, at least in-part, sourced from wild populations in neighbouring countries.

### 4.5. Source

Markets are central to trade in wildlife for *muthi* purposes, with healers as major purchasers [21]. Interestingly, some healers also stated that they sourced parts from game reserves and lion breeders (i.e., captive-bred lions). Captive-bred lion parts being directly sold and donated for use in *umuthi* was noted by [5,8,16,62]. We did not explore exactly what respondents understood ‘game reserves’ to be. However, we note that there are a variety of land use types in Southern Africa colloquially referred to in this way ranging from state land and national parks to private commercial properties for tourism, hunting, and ranching. Traders stated that they bought lion parts from ‘people’; the cryptic nature of this response may indicate that they were attempting to conceal the precise origins of material. In lower proportions of responses, traders indicated poachers and gatherers as categories that they bought lion parts from. It is likely that responses referring to illegal activity are under-represented. Nevertheless, our results indicate that an uncertain proportion of traded lion in *muthi* markets in South Africa is likely to come from poached sources. Traders also indicated that they obtained lion parts from game reserves and our results indicate that workers on reserves are a likely conduit for lion parts to some traders. Interestingly, captive-breeding facilities or ‘farms’ were not cited by any of the traders surveyed as a source whereas healers did—however, [5,8] note that a lion skin observed in a South African market was allegedly supplied by a captive facility. We believe that some captive-breeders have established personal relationships with traditional healers to whom they donate or sell lion parts (noted in [16]). 

Amongst traders, Zimbabwe was indicated as a source of lion parts, along with lesser proportions of responses mentioning Zambia, Eswatini (Swaziland), and Mozambique. Body-part removals from lion mortalities in Zimbabwe’s Hwange landscape appear to be largely linked to traditional uses of lion parts [4], and in Limpopo National Park, Mozambique, body-part removal and trade has been suggested to incentivize lion killings [61]. An active domestic trade in lion products has been previously recorded between Southern African countries [5]. Our results indicate that the footprint of South African *muthi* trade in lion parts extends beyond the borders of South Africa. However, the exact extent and impacts are as-yet unknown. Nevertheless, culturally motivated trade and use is a largely under-considered anthropogenic threat to African carnivores [5,7].

### 4.6. Supply

Our results indicated that a majority of traders and healers were finding it harder to get lion parts. One trader (TR9) indicated that it was issues in supply and stated that they obtained lion parts from workers from game reserves. We cannot generalise the responses of one trader and do not wish to unduly speculate, however, we note the continuing decline in the captive-bred lion industry and the unknown impact of possible cascades of supply and demand in lion parts [62]. Our results indicate that *muthi* trade may be more deserving of detailed consideration as part of the extensive captive-bred lion trade system. 

There were substitutes for lion identified by our respondents, however the transition of substitutes into real world practice is well-acknowledged to be difficult [4,63,64,65]. Interestingly, traders suggested a substitute, *umkhulekelwa* (a plant), that healers did not. We cannot identify what species of plant *umkhulekelwa* is with any certainty, but it is associated with a meaning of reverence, or prayers to a higher-being and facilitation of communication with ancestors (pers. obs.). It was not mentioned by healers despite being referred to as a healing ingredient (pers. obs.). It is possible that healers did not feel at liberty or were unwilling to reveal what *umkhulekelwa* is or may not have viewed it as an important substitute. The plant *umdlebe* was also mentioned as a potential substitute. *Umdlebe* is typically *Euphorbia cupularis* (i.e., dead-man’s tree; a much-feared tree with very poisonous sap), but from personal observation this also includes *Pterocarpus* spp. (i.e., bloodwood; mechanical injury to the stem resembles a bleeding, disfiguring wound). More healers said that there were possible substitutes for lion than said there were not. However, the proportion that said there were no suitable substitutes was sizeable (35%), and indicates that, for at least some practitioners, lion occupies an immutable role. Suggested substitutes appeared to follow the philosophy of the Doctrine of Signatures [22]; with other animals and plants associated with strength, power, fear, danger, and bewitchment (e.g., snakes, big cats and dangerous game, *Encephalartos* spp., *E. cupulare,* and *Pterocarpus* spp.) being suggested. Substitutions are likely to be context specific, as a single species can have multiple symbolic meanings which may vary between parts of the organism and ancestral spirits being invoked. In addition, ethnotaxonomy (including the nomenclature of *muthi* species) is well-recognised to be complex, with the same or similar names sometimes applied to multiple species, often united by taxonomic, trait, habitat, or symbolic similarity [66,67,68,69,70]. Substitution for lion *umuthi* is therefore likely to be complex and is not well understood and we urge caution in the interpretation of our results. In-depth investigation of substitutions was outside the scope of this study, but we highlight a need for further exploration of the dynamics of substitution in *muthi* practice and caution against simplistic interpretations.

We raise concern over suggestions to transition traditional medicine from animals to plant substitutes (e.g., [15]) without due consideration of the potential conservation impacts upon plant species (e.g., threatened cycads [*Encephalartos* spp.] [71,72]), and caution against the prioritisation of animals over plants in conservation thought [73]. 

We highlight the need to monitor a wide range of species in traditional medicinal systems in order to best identify cascading impacts of changing supply and demand. In particular, we are mindful of South Africa’s recent decision to transition away from captive-lion breeding and hunting. We make no comment on the rightness of this decision. However, the potential contribution of captive-bred lions to traditional cultural practice in South Africa has been largely overlooked. Further detailed research is required into the role of wild and captive-bred lion in cultural practice in South Africa and potential impacts on wild populations. We highlight cultural systems as users of lion products and emphasize that traditional users have been thus-far silent stakeholders in decisions concerning captive-bred lion farming in South Africa. There is therefore a pressing need for better integration of traditional users of lion products into conservation decision-making and policy forums in South Africa. 

### 4.7. Sustainability

The majority of traders and healers believed lion populations in South Africa to be decreasing—in contrast to the current state of lion populations which are stable or increasing [1]. It is possible that respondents confused range-wide status with the situation in South Africa [1]. It is also possible that their perceptions were based on changing availability of parts in South Africa, which were said to be becoming harder to source. Understandably, loss of business was a primary concern related to decreasing lion availability, but loss of culture only featured with a minority of respondents: suggesting that livelihood concerns dominated the worries of stakeholders in this system. Traders desired greater access to carcasses, and lion-containing land areas (we lack detail on the specific designations of land use types), whilst healers favoured breeding farms which they had identified as sources of parts. Interestingly, law enforcement was the most frequently cited protection measure. This was not expected as actors in the lion *muthi* trade system often operate outside of legal trade and were found to be distrusting of law enforcement (pers. obs.). Indeed, a number of respondents stated that they sourced lion parts from poachers. Unsurprisingly, none of these individuals proffered law enforcement as a means to ensure future access to lion products. Two individuals sourced material from abroad—in contravention of the Convention on International Trade in Endangered Species of Wild Fauna and Flora (CITES; [14]). Amongst some respondents there is thus an apparent paradox between their own law-breaking activities and their suggestion for the future of lion in South Africa. We did not ask whether they desired a future supply of lion parts, neither did we explore respondent understanding of the law or any trade-offs regarding livelihoods and cultural practice against compliance with the law. Future studies could usefully seek to explore traditional trader and user interactions with law enforcement and legislation.

## 5. Conclusions

In this study we set out to gain a better understanding of the role of lion within a contemporary traditional medicine system (*muthi*) in South Africa and explore trade dynamics of lion parts in two major South African traditional wildlife markets. We stress the cultural importance of lion in traditional medicinal and cultural practices, which is probably far in excess of its absolute parts-sales and livelihood contribution. Indeed, the complex and interlinked nature of traditional medicine indicates that there is limited utility in considering items as ‘stand-alone’ without acknowledging their wider context. Although we found that certain body parts (i.e., mainly from paws) dominated stated demand and sales, lion cannot be considered, in respect of its role in *umuthi*, in the singular. Rather, we emphasize that, to traders and practitioners, a lion represents a variety of parts, ingredients, and symbols. A more complete understanding of the cultural role of lion in South African traditional medicine will hinge on fuller exploration and acknowledgement of its multifaceted nature. A key outcome of this work is a greater understanding of the guidance of ancestral spirits in the use, and trade, of lion parts for *umuthi*. Although a spiritual aspect of *umuthi* is widely accepted by the conservation community, the significance, and dynamics of spiritual guidance in the use of wildlife products, including lion, have not been acknowledged, much less explicitly explored. We therefore introduce the conception of ancestral spirits as a form of stakeholder and ‘gatekeepers’ in the traditional lion *umuthi* system (Figure 8) and suggest that further work seeks to better determine how spiritual practice drives lion part trade. 

The impact of traditional trade on wild lion populations remains largely under-considered and unquantified. Nevertheless, our results indicate that South African *muthi* trade in lion parts has a trade footprint that reaches internationally within Southern Africa. In order to protect wild lion populations from illegal exploitation, we highlight the need for effective and socially just law enforcement and trade regulation engagement with stakeholders in culturally traditional lion trade systems.

## Figures and Tables

**Table 1:** The degree of similarity of lion parts cited between and within respondent groups, expressed as the number of shared parts and the Sørenson’s similarity index (SSI) percentage of shared parts.

	(a) Similarity between Respondent Groups			(b) Similarity within Respondent Groups	
	Traders (*n* = 10): Parts They Sell *	Traders (*n* = 7): Parts in Demand **	Healers (*n* = 20): Parts for Themselves	Number of Parts Similar	% Parts Similar
Traders (*n* = 10): parts they sell				3.4 ± 2.9	40% ± 32%
Traders (*n* = 7): parts in demand	12 (59%)			1.8 ± 1.7	35% ± 30%
Healers (*n* = 20): parts for themselves *	18 (77%)	12 (80%)		3.0 ± 1.9	46% ± 25%
Healers (*n* = 11): parts for patients *	19 (79%)	12 (77%)	16 (87%)	2.1 ± 1.1	51% ± 30%

* When combining the two healer categories (parts purchased for themselves and patients) to get a value for all the parts healers purchase, there are 20 parts in common with parts the traders sell (82% similar). ** When combining the two healer categories (parts purchased for themselves and patients) to get a value for all the parts healers purchase, there are 12 parts in common with parts the traders say are in demand (75% similar)

## Data Availability

The data and code that support the findings of this study are openly available in ZivaHub, a UCT-based Figshare repository at https://doi.org/10.25375/uct.19382591 (accessed on 3 March 2022).

## Supplementary Materials

The following supporting information can be downloaded at: https://www.mdpi.com/article/10.3390/ani12223169/s1, Supplementary Document S1: Informed consent form. Supplementary Document S2: Survey questionnaire.
