# Supplementary material for: Contemporary Cultural Trade of Lion Body Parts
Source: Animals (Basel). 2022 Nov 16;12(22):3169. doi: 10.3390/ani12223169 (PMC9686618; doi:10.3390/ani12223169)

SUPPLEMENTARY MATERIALS

Supplementary Document S1. Informed consent form used for the study on the cultural value of lions among traditional healers (*n* = 20) and traders (*n* = 10) in South Africa.

Informed voluntary consent clause to participate in this research study

Study title: Cultural value and sustainability of wildlife trade among traditional healers and muthi traders in South Africa.

Invitation to participate and benefits: You are invited to participate in a research study conducted with traditional healers and traders. This proposed research project aims to investigate the trends of traditional healing practices, the muthi trade and the perceptions about conservation of wildlife among traditional healers and muthi markets traders in South Africa. We believe that your experience would be a valuable source of information and hope that by participating you may gain useful knowledge, as well as have an opportunity to share your interests and concerns regarding this species, its trade and the value of alternatives as a direct consumer.

Procedures: During this study, you will be asked to answer some questions regarding your use lion parts as a traditional healer or trader, how society perceives this species and your knowledge about the trade of lion parts. I propose to sample between 25–30 healers and 10% of the muthi traders per study site using a semi-structured questionnaire. Selection will be based on referrals from existing traditional healer organisations. I will attempt to interview as wide a range of respondents and healer organizations as possible. The questionnaire will include questions on: 1) animal species used, 2) the demand and difficulty in obtaining different species, 3) the role ancestors play in selecting an animal or part thereof to be used, 4) where healers obtain animal material for use 5) how frequent the trader/healer buys material (weekly/monthly/yearly) and 6) what has influenced the changes and trends in material traded in markets.

Recording: We will keep written or audio records as part of the study. If you object, please tell us and we will not keep a formal record of our interaction.

Risks: There are no potentially harmful risks related to your participation in this study.

Withdrawal disclaimer: Your participation is completely voluntary; you may refuse to participate and you may withdraw at any time without having to state a reason and without any prejudice or penalty against you. Should you choose to withdraw, the researcher commits not to use any of the information you have provided. Note that the researcher may also withdraw you from the study at any time.

Confidentiality: All information collected in this study will be kept private in that you will not be identified by name or by affiliation to an institution. Confidentiality and anonymity will be maintained as pseudonyms will be used. None of the data collected may be used in any legal action against you.

What agreeing means: By agreeing to this consent form, you agree to participate in this research study. The aim, procedures to be used, as well as the potential risks and benefits of your participation have been explained verbally to you in detail, using this form. Refusal to participate in or withdrawal from this study at any time will have no effect on you in any way. You are free to contact us, to ask questions or request further information, at any time during this research.

Supplementary Document S2. Survey questionnaire.

Interview ID: ______________________ *Date:* ______________

Province: ______________________ *Locality:* ______________

Cultural value of lions: *Muthi traders and healers survey*

TRADERS AND SHOP OWNERS ONLY

T1 Do customers ask for lion parts or do you recommend them?

Ask	Recommend	Both	Other

T2 Do you sell parts alone or as mixed medicine?

Alone	Mixed	Both	Other

T3 If medicine, is it mixed with other animal parts, plant material or both?

Animals	Plants	Both	Other

T4 If they are mixed, please name the organisms:

Open with prompt

T5 Is there a customer preference between male and female lion parts?

Male	Female	None	Uncertain	*Other*

T6 If there is, what is the reason for the preference?

Open with prompt

T7 Requests for lion parts are…?

Increasing	Same	Decreasing	Uncertain	*Other*

T8 Please explain why?

Open with prompt

T9 Which lion parts do you sell? *- Select*

A - *Face*	B - *Head*	C - *Mane*	D – *Tail*	E - *Back paws*	F - *Front paws*	G - *Claws*
H - *Teeth*	I - *Skin*	J - *Fat*	K - *Spine + tail*	L - *Back legs*	M - *Tarsal*	N - *Kneecap*
O - *Ribs*	P - *Scapula*	Q - *Front legs*	R - *Carpels*	S - *Skull + jaw*	*Other*	

T10 Are certain parts of the lion more in demand than others? *- Select + rank*

A - *Face*	B - *Head*	C - *Mane*	D - *Tail*	E - *Back paws*	F - *Front paws*	G - *Claws*
H - *Teeth*	I - *Skin*	J - *Fat*	K - *Spine + tail*	L - *Back legs*	M - *Tarsal*	N - *Kneecap*
O - *Ribs*	P - *Scapula*	Q - *Front legs*	R - *Carpels*	S - *Skull + jaw*	*Other*	

T11 Which ones sell for the highest and lowest prices? *- Select + rank*

A - *Face*	B - *Head*	C - *Mane*	D - *Tail*	E - *Back paws*	F - *Front paws*	G - *Claws*
H - *Teeth*	I - *Skin*	J - *Fat*	K - *Spine + tail*	L - *Back legs*	M - *Tarsal*	N - *Kneecap*
O - *Ribs*	P - *Scapula*	Q - *Front legs*	R - *Carpels*	S - *Skull + jaw*	*Other*	

Notes:

Cultural value of lions: *Muthi traders and healers survey*

DIVINERS AND HERBALISTS ONLY

H1 Are lion parts used for rituals during training?

Yes	No	Uncertain	Other

H2 If yes, for which ancestral spirit?

Open with prompt

H3 Rank who you buy for most often? *(Most often 1, least often 3)*

Yourself	Customers	Both	Other

H4 If for yourself, what is it for?

Divination sets	Personal use	Both	*Other*

H5 Is there a customer preference between male and female lion parts?

Male	Female	None	Uncertain	*Other*

H6 If there is, what is the reason for the preference?

Open with prompt

H7 Which parts do you usually buy for yourself? *- Select*

A - *Face*	B - *Head*	C - *Mane*	D – *Tail*	E - *Back paws*	F - *Front paws*	G - *Claws*
H - *Teeth*	I - *Skin*	J - *Fat*	K - *Spine + tail*	L - *Back legs*	M - *Tarsal*	N - *Kneecap*
O - *Ribs*	P - *Scapula*	Q - *Front legs*	R - *Carpels*	S - *Skull + jaw*	*Other*	

H8 Are certain parts of the lion more in demand than others? *- Select + rank*

A - *Face*	B - *Head*	C - *Mane*	D - *Tail*	E - *Back paws*	F - *Front paws*	G - *Claws*
H - *Teeth*	I - *Skin*	J - *Fat*	K - *Spine + tail*	L - *Back legs*	M - *Tarsal*	N - *Kneecap*
O - *Ribs*	P - *Scapula*	Q - *Front legs*	R - *Carpels*	S - *Skull + jaw*	*Other*	

H9 Which ones sell for the highest and lowest prices? *- Select + rank*

A - *Face*	B - *Head*	C - *Mane*	D - *Tail*	E - *Back paws*	F - *Front paws*	G - *Claws*
H - *Teeth*	I - *Skin*	J - *Fat*	K - *Spine + tail*	L - *Back legs*	M - *Tarsal*	N - *Kneecap*
O - *Ribs*	P - *Scapula*	Q - *Front legs*	R - *Carpels*	S - *Skull + jaw*	*Other*	

H10 Why are lions important to your practice?

Open with prompt

Notes:

Cultural value of lions: *Muthi traders and healers survey*

ALL RESPONDENTS

A1 How do you obtain the lion parts?

Open with prompt

A2 Where do the lion parts come from?

Open with prompt

A3 Describe the cultural value of lions?

Open with prompt

A4 Lion numbers in South Africa are?

Increasing	Stable	Decreasing	Uncertain	*Other*

A5 If lion numbers were/are decreasing, how would that affect your business/practice?

Open with prompt

A6 Are there substitutes that can be used when lion parts cannot be obtained?

Yes	No	Uncertain	*Other*

A7 If yes, please list them (and the relevant parts) – If no, explain why not?

Open with prompt

A8 What do you think motivates the use of lion products?

Open with prompt

A9 Is getting lion parts…?

Easier	Same	Harder	Uncertain	*Other*

A10 Please explain?

Open with prompt

A11 Do you prefer wild lion parts? *(Note if they know about captive breeding or not)* *Yes* *No*

Yes	No	Indifferent	*Other*

A12 Please explain why?

Open with prompt

A13 What do you think can be done to ensure lion parts are available in the future?

Open with prompt

Notes:

Questionnaire graphic accompanying questionnaire and sent via WhatsApp to persons completing the survey telephonically. Graphic applies to questions T9–T11 and H7–H9.

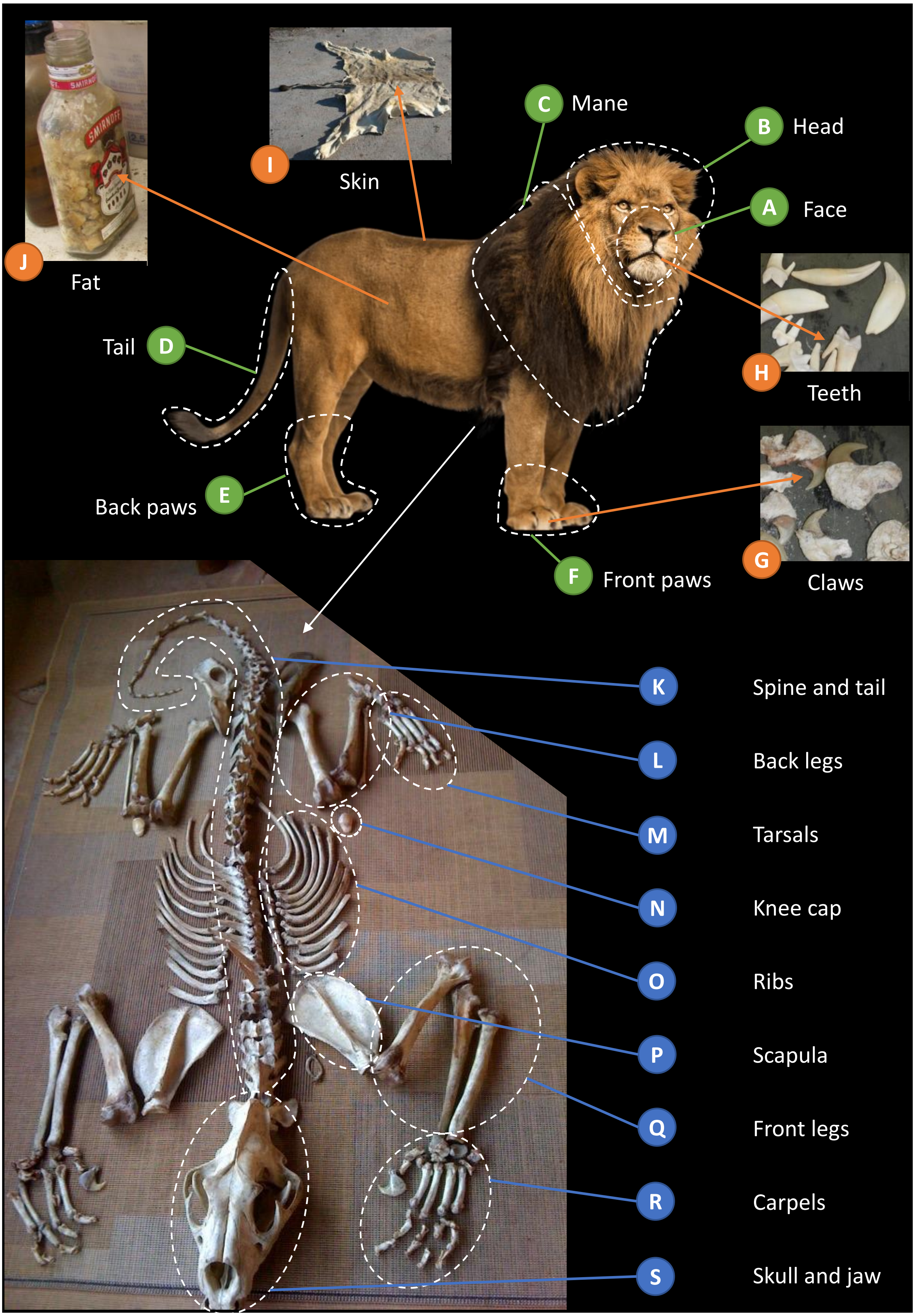

Supplement: Supplementary file 1 [file animals-12-03169-s001.zip › animals-1974822-supplementary.pdf]
